# Sulfur Metabolism as a Promising Source of New Antifungal Targets

**DOI:** 10.3390/jof8030295

**Published:** 2022-03-13

**Authors:** Jorge Amich

**Affiliations:** 1 Mycology Reference Laboratory, National Centre for Microbiology, Instituto de Salud Carlos III (ISCIII), Majadahonda, 28222 Madrid, Spain; jamich@isciii.es; 2 Manchester Fungal Infection Group (MFIG), Division of Evolution, Infection, and Genomics, Faculty of Biology, Medicine and Health, University of Manchester, Manchester M13 9NT, UK

**Keywords:** *Aspergillus fumigatus*, sulfur metabolism, antifungal targets

## Abstract

Fungal infections are a growing threat to human health. Despite their clinical relevance, there is a surprisingly limited availability of clinically approved antifungal agents, which is seriously aggravated by the recent appearance and fast spread of drug resistance. It is therefore clear that there is an urgent need for novel and efficient antifungals. In this context, metabolism is recognized as a promising source for new antifungal targets and, indeed, there are new drugs in development that target metabolic pathways. Fungal sulfur metabolism is particularly interesting, as many of its processes are essential for viability and/or pathogenicity and it shows substantial differences with human metabolism. This short-review will summarize our current knowledge of sulfur-related genes and routes that are important for *Aspergillus fumigatus* virulence, which consequently could be pursued for drug development.

## 1. Introduction

*Aspergillus fumigatus* is a ubiquitously distributed, saprophytic filamentous fungus. In nature, it commonly undergoes an asexual cycle, which produces a profusion of airborne uninucleate spores, called conidia. As a result of its high abundance and wide distribution, it is believed that every human inhales hundreds or even thousands of these conidia on a daily basis, which, due to their small size (2–3 µm), can reach deep into the respiratory tract [1,2]. This has normally no negative consequences, as the orchestrated action of the mucociliary, the respiratory epithelium and the innate immune system, very efficiently control and eliminate the spores [3,4,5]. However, individuals with immune imbalances and/or immunosuppression are at risk of developing aspergillosis infections. Aspergillosis is a general term to denote a spectrum of respiratory diseases, which can be broadly classified in three major subtypes: allergic, chronic and invasive [6,7]. It is not the aim of this short review to enter into details about the different types of aspergilloses, yet it is important to mention that a common characteristic of all of them is the germination and (some level) growth of *Aspergillus* spp. (being *A. fumigatus* the most common etiological agent) in the respiratory tract. Consequently, invasive and chronic aspergilloses are always treated with antifungals, and even allergic aspergilloses are sometimes treated with these drugs.

At the moment of publication of this short review, there are only three classes of antifungals in clinical use to treat deep aspergillosis infections: azoles, polyenes and echinocandins. All of them suffer from pharmacological drawbacks, including poor bioavailability, drug–drug interactions, or toxicity [8,9]. Despite the availability of drugs, the mortality associated with *A. fumigatus* infections remains unacceptably high: 38% 5-year mortality rate for chronic pulmonary aspergillosis [10] and ranging in invasive aspergillosis from ~50% in early diagnosed and treated patients to nearly 100% if diagnosis is missed or delayed [11]. Moreover, in *A. fumigatus,* resistance to the first line therapeutics, triazole drugs, has emerged and seems to be increasing all over the world, causing serious problems for the clinical management, as the therapeutic options are reduced and the mortality rates caused by resistant isolates are higher [10,12,13]. Despite this, there is hope on the horizon, as several new and promising antifungal drugs are in development, a few of them in phase II and III clinical trials (for a complete review see [14]). Some drugs have the same targets, yet have superior characteristics compared to current agents, and some have completely new mechanisms of action. This promising perspective is only possible as a virtue of the intensive research performed by the fungal community in recent years, at all levels, basic, translational and clinical. Nevertheless, we should not be content and relax. We should continue, discovering new targets and drugs to make sure that we always have working antifungals approved and promising agents in development.

In this respect, this review summarises the work we have performed in recent years to characterise *A. fumigatus* sulfur metabolism. Metabolism has been recognized as a promising source of antifungal targets, as it is a fundamental aspect of fungal physiology and vitality and is known to be crucial for pathogenicity. Sulfur metabolism is of special interest as it comprises essential metabolic routes that are not well-conserved in host cells and, therefore, provide prospects for specific antifungal targets and therapies.

## 2. Overview of Fungal Sulfur Metabolism

**Figure 1:**
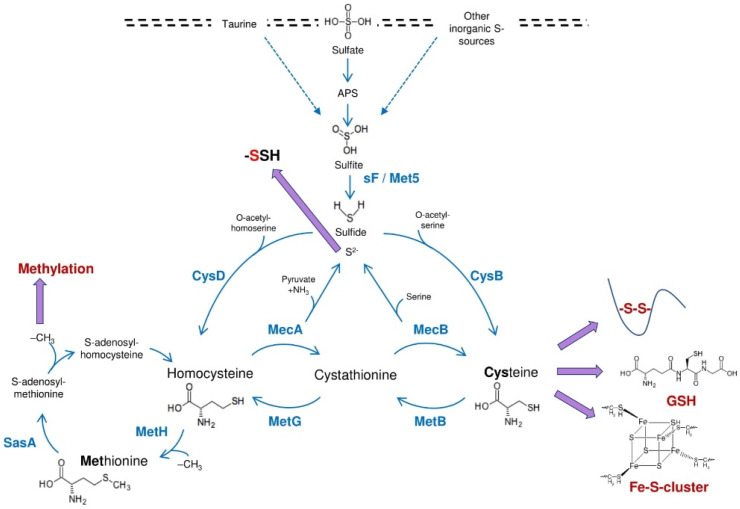
Overview of *Aspergillus fumigatus* sulfur metabolism.

Sulfur is an essential macronutrient, and as such, *A. fumigatus* must obtain it from the environment to thrive and grow. Sulfur is a constituent of the S-containing proteinogenic amino acids cysteine and methionine, and of essential organic molecules such as coenzyme-A, glutathione, iron-sulfur (Fe-S) clusters and S-adenosylmethionine (which donates the methyl group in the methylation process) (Figure 1). Inorganic sulfur compounds are taken up and reduced intracellularly to sulfite (SO_3_^2+^) [15]. The best characterised route is the sulfate (SO_4_^2+^) assimilation pathway, as it is a preferred S-source for fungi [16]. Sulfate assimilation occurs via a well-defined mechanism involving its uptake by specialised permeases, activation by ATP-driven phosphorylation, reduction to sulfite, and further reduction to sulfide (S^2−^). The latter is condensed with O-acetyl serine to yield cysteine, or with O-acetyl homoserine to yield homocysteine, from which methionine can be formed [16] (Figure 1). The route of interconversion between cysteine and methionine is called the trans-sulfuration pathway and is the core of fungal sulfur metabolism.

The assimilation of sulfate, other inorganic S-sources and taurine converge in sulfite, which is further reduced to sulfide and incorporated into the metabolism. Cysteine and methionine serve as important constituents of essential processes, as protein folding (disulfide bonds), glutathione (GSH), iron-sulfur clusters (Fe-S) and *S*-adenosylmethionine-dependent methylations. These amino acids can be interconverted via the trans-sulfuration pathway. Sulfide generated in the pathway can exert the post-translational modification persulfidation.

sF/Met5 = sulfite reductase; CysB = cysteine synthase; CysD = homocysteine synthase; MecA = cystathionine-β-synthase; MecB = cystathionine-Ɣ-lyase; MetB: cystathionine-γ-synthase; MetG: cystathionine-β-lyase; MetH: = methionine synthase; SasA = *S*-adenosylmethionine syntethase.

## 3. The Regulation of Sulfur Assimilation Is Crucial for *A. fumigatus* Virulence

In our initial work, we characterised MetR [17], a bZIP transcription factor orthologue to the *A. nidulans,* master regulation of sulfur metabolism [18]. We showed that a *ΔmetR* deletion mutant was unable to grow on any of the tested inorganic S-sources, on some organic S-sources and on complex S-sources. Transcriptomic investigations, both by RT-PCR and RNA-seq, indicated that MetR is involved in the regulation of sulfur metabolic genes, particularly related with sulfur assimilation, and links sulfur metabolism and iron homeostasis. Importantly, we demonstrated that MetR is crucial for *A. fumigatus* virulence. In a leukopenic model of invasive pulmonary aspergillosis (IPA), *ΔmetR* had a strong reduction in virulence, as shown by an >80% survival of infected mice and a competitive index <0.1 in an in vivo competition assay with the wild-type strain. Therefore, targeting MetR can be considered a promising strategy to fight aspergillosis infections. Targeting transcription factors for antifungal development is an underappreciated option, which is nevertheless supported by the great relevance for virulence of some of them and the recent technical advances that make this a real possibility [19]. Moreover, RNA-based therapeutics, which could silence the expression of key transcriptional factors, are advancing quickly and have been recently highlighted as a promising strategy for the future of antifungal treatment [20].

## 4. The Assimilation of Inorganic Sulphur Sources Is Dispensable for Growth in the Tissues, Whilst Cysteine Biosynthesis Is Important for Virulence

Having demonstrated that a proper regulation of sulfur assimilation is crucial for virulence, we aimed to determine the sulfur source(s) that *A. fumigatus* exploits during intrapulmonary growth. To this aim, we deleted the β-subunit of the sulfite reductase-encoding gene, *sF* [21]. As expected, (Figure 1), a *ΔsF* mutant was unable to grow on any of the tested inorganic S-sources (except S^2-^) or on taurine, yet grew well on other organic S-sources. Despite this clear and strong phenotype, the mutant was completely virulent in a leukopenic murine model of IPA. Therefore, the assimilation of inorganic S-sources and taurine is dispensable for virulence and the related pathways do not seem to be good drug target candidates.

We next endeavoured to construct a cysteine auxotroph to examine if *A. fumigatus* can acquire this amino acid from the tissues. We constructed a double *ΔcysBΔmecA* mutant to block both the direct and alternative pathways of cysteine biosynthesis (Figure 1) [21]. As expected, the double mutant was only able to grow when cysteine was present in the medium. Interestingly, the cysteine auxotroph had a significantly reduced virulence in a leukopenic model of IPA, indicating that the amount of readily available cysteine in the lung tissues is limited, and therefore that cysteine is not the major S-source exploited in the lungs. Importantly, we showed that the cysteine auxotroph was not more sensitive to oxidative stress and was killed at a similar level as the wild type by bone marrow derived neutrophils [21], suggesting that the decreased virulence of the *ΔcysBΔmecA* mutant is not due to a higher susceptibility to host derived oxidative stressors. In conclusion, targeting cysteine biosynthesis could also be a valuable strategy to fight aspergillosis infections.

## 5. Methionine Synthase Is Essential for *A. fumigatus* Growth and Virulence

Simultaneously to the construction of the cysteine auxotroph, we attempted to delete the methionine synthase encoding gene, *metH*, to derive a methionine auxotroph. However, we found that this gene was essential for *A. fumigatus* viability [20], which was not such a surprising result, as its essentiality had already been reported in *Candida albicans* and *Cryptococcus neoformans* [22,23]. Given this conservation of essentiality in important fungal pathogens, and the fact that the human and fungal methionine synthases belong to different families (cobalamin-dependent EC 2.1.1.13 the former and cobalamin-independent EC 2.1.1.14 the latter), we believed that it was warranted to study the potential of methionine synthase as an antifungal target. We firstly investigated the underlying reason for *metH* essentiality beyond methionine auxotrophy. We found that the absence of methionine synthase triggered a metabolic imbalance, likely due to a forced flow of glucose through the pentose phosphate pathway, that causes a decrease in cell energetics [24]. Using the *tetOFF* conditional promoter, we observed that the downregulation of *metH* in 8 h germinated conidia, as well as in 12 and 16 h-grown mycelia, sustainably halted fungal growth. We then established in vivo models of established infection, where we downregulated *metH* expression after the initiation of infection. For these experiments, we constructed a *cyp51A_tetOFFΔcyp51B* strain to serve as control, as we reasoned that any new target in perspective should be compared to the target of the gold-standard treatment, the azoles. In *Galleria mellonella,* the downregulation of *metH* after 6 h of infection resulted in a significant decrease in mortality, similar to that obtained by downregulating *cyp51A*. In mice, we found that the high doses of doxycycline required to downregulate fungal gene expression in the lungs were toxic to the animals, thus, we could not evaluate the effect on survival. Yet, we could show that the downregulation of *metH* produced a significant reduction in fungal burden, again similar to the reduction obtained when downregulating *cyp51A* [24]. Finally, we performed a structure-based virtual screening demonstrating that fungal methionine synthases have exclusive pockets that can be specifically targeted. Therefore, fungal methionine synthases are really promising antifungal targets, worth the effort to pursue for drug development

## 6. Persulfidation Is Relevant for *A. fumigatus* Pathogenic Potential

Persulfidation is a post-translational modification in which an activated S^2-^ attacks thiol (-SH) groups of cysteine residues in target proteins to form persulfide groups (-SSH) [25]. Persulfidation can increase or decrease the specific activity of the modified proteins and, consequently, it has a prominent effect on cellular metabolism and physiology [26,27]. Although the exact mechanism that activates H_2_S is still under study, three enzymes that function in sulfur metabolic routes have been shown to also be able to generate H_2_S and be related with the formation of persulfide groups: cystathionine β-synthase (CBS), cystathionine γ-lyase (CTH), and 3-mercaptopyruvate sulfurtransferase (MST) [28,29]. We constructed the three single deletion mutants in *A. fumigatus* and corroborated that two of them, *ΔmecA* (CBS) and *ΔmecB* (CTH), had significantly reduced levels of persulfidation [30]. Importantly, those mutants grew normally on all tested S-sources, suggesting that the metabolic function of the enzymes is not crucial for the stability of the trans-sulfuration pathway. We were unable to construct a double mutant, which led us to propose that persulfidation may be an essential cellular process that cannot be fully disrupted. Compared with the wild type, the *ΔmecB* mutant was killed to a higher degree by human and murine host effector cells (including macrophages, neutrophils and epithelial cells) and had significantly reduced virulence in two murine models, leukopenic and corticosteroid, of invasive pulmonary aspergillosis [30]. Therefore, correct levels of protein persulfidation are important for *A. fumigatus* pathogenic potential and, consequently, tampering with this process may be a good approach to develop novel antifungal strategies.

## 7. Many Sulfur-Derived Molecules Are Fundamental for *A. fumigatus* Physiology and Virulence

After its assimilation in the metabolism, sulfur derives in a variety of different molecules that are important for *A. fumigatus*’ viability and/or virulence. For instance, an auxotroph of the S-containing vitamin thiamine showed reduced virulence in murine models [31]. A mitochondrial acetyl-CoA acetyltransferase has been described to be a potential antifungal target [32]. *S*-adenosylmethionine is involved in all cellular methylations, and accordingly, the synthetase has been shown to be essential for *A. nidulans* viability [33], although its function in *A. fumigatus* has not been tested yet. Furthermore, sulfur is fundamental for cellular redox homeostasis, as it is a fundamental constituent of several important systems. Sulfur is key for the activity of the molecules ergothioneine and glutathione, which also interact with the toxin gliotoxin, the biogenesis of which requires sulfur [34], an *A. fumigatus’* important virulence factor [35,36]. Sulfur is also crucial in peroxiredoxins, which have been described to be important for *A. fumigatus* virulence [37,38] and in thioredoxins, another redox system relevant for virulence [39]. Finally, biogenesis of iron-sulfur cluster is an essential process for viability [40], and sulfur is involved in iron sensing via the glutaredoxin GrxD [41].

## 8. Conclusions and Perspectives

This short review has highlighted various sulfur-related processes which are important for *A. fumigatus* pathogenicity and therefore could be good targets for antifungal drug development. Yet, there are still many proteins and processes that are potential candidates, as sulfur metabolism seems to be a profuse source of prospective targets. Methionine synthase is particularly promising, as we have shown that its downregulation in established infections conferred a beneficial effect comparable to the downregulation of the target of azoles, the gold-standard treatment for aspergillosis infections.

This knowledge is available now for the community to design and/or screen for molecules that target these candidates, to hopefully develop novel antifungal agents. The drug development path is long but starting with a well-validated target is a good foundation for the process [42,43].

## Data Availability

Not applicable.
